# A Systematic Review of the Association Between Muscular Fitness and Telomere Length Across the Adult Lifespan

**DOI:** 10.3389/fphys.2021.706189

**Published:** 2021-07-15

**Authors:** Adilson Marques, Miguel Peralta, Priscila Marconcin, Duarte Henriques-Neto, Élvio Rúbio Gouveia, Gerson Ferrari, João Martins, Hugo Sarmento, Andreas Ihle

**Affiliations:** ^1^CIPER, Faculty of Human Kinetics, University of Lisbon, Lisbon, Portugal; ^2^ISAMB, University of Lisbon, Lisbon, Portugal; ^3^Faculty of Human Kinetics, University of Lisbon, Lisbon, Portugal; ^4^Departamento de Educação Física e Desporto, Universidade da Madeira, Funchal, Portugal; ^5^Interactive Technologies Institute, LARSyS, Funchal, Portugal; ^6^Laboratorio de Ciencias de la Actividad Física, el Deporte y la Salud, Facultad de Ciencias Medicas, Universidad de Santiago de Chile, Santiago, Chile; ^7^University of Coimbra, Research Unit for Sport and Physical Activity (CIDAF) Faculty of Sport Sciences and Physical Education, Coimbra, Portugal; ^8^Center for the Interdisciplinary Study of Gerontology and Vulnerability, University of Geneva, Geneva, Switzerland; ^9^Swiss National Centre of Competence in Research LIVES—Overcoming Vulnerability: Life Course Perspectives, Lausanne and Geneva, Switzerland; ^10^Department of Psychology, University of Geneva, Geneva, Switzerland

**Keywords:** handgrip, physical fitness, leukocyte, genetics, muscle

## Abstract

This study aimed to systematically review the association between telomere length (TL) and muscular fitness. In October 2020, an articles search was applied to PubMed, Scopus, and Web of Science. Eligibility criteria included: cross-sectional, prospective, and experimental study design; outcomes included TL; results expressed the relationship between muscular fitness and TL; studies published in English, Portuguese, or Spanish. Nine studies were included in the review. Results from the four prospective studies are mixed. In one study, the changes in TL were associated with grip strength. Another study concluded that longer mid-life TL was associated with increased grip strength later in life. However, in the other two studies, the association between TL and sarcopenia was not strong. Nevertheless, longer TL was associated with a slower decline in grip strength in older people. From the four cross-sectional studies, three indicated that TL was associated with muscular fitness. On the other hand, in a study with powerlifters, TL remained within the range of values found in subjects with no history of regular strength training, supporting the notion that muscular fitness was not associated with TL. The cross-sectional and prospective studies showed that the relationship between TL and muscular fitness is not conclusive. It seems that there is a positive association between TL and muscular fitness in middle-aged and older adults. However, among younger adults, this relationship was not observed.

## Introduction

Chromosome ends are protected by tandem repeats of hexanucleotide units named telomeres (Lu et al., 2013). Telomeres are critical in regulating cellular replicative capacity (Codd et al., 2013). The functional status of telomeres depends on the telomeric nucleoprotein structure's stability and length (Wang et al., 2018). The telomeres shorten each time a cell divides because of the inability of the DNA polymerase to replicate the ends of the linear molecules completely. Telomere length (TL) shortening is associated with cellular senescence (Liu et al., 2019), oxidative stress (Mundstock et al., 2015a), increased inflammatory process (Arsenis et al., 2017), tobacco smoking, alcohol consumption, and physical activity (Mather et al., 2010; Codd et al., 2013; Lin et al., 2019). Also, psychological disorders and low social-economic levels can accelerate the TL shortening process (Zhang et al., 2014). However, TL shortening is not an irreversible process because of telomerase, an enzyme capable of extending telomeres. Thus, it seems likely that TL is subject to different regulations in body systems to maintain tissue integrity (Kadi et al., 2008). TL in skeletal muscle can be considered more dynamic structures under the influence of the environment, such as physical activity, exercise, and physical fitness (Kadi and Ponsot, 2010).

Physical activity and physical fitness appear to play an essential role in the TL shortening process (Soares-Miranda et al., 2015; Tucker, 2017). Several potential mechanisms are involved, including changes in telomerase activity, oxidative stress, inflammation, and decreased skeletal muscle satellite cell content (Arsenis et al., 2017). Oxidative stress is improved by physical activity (Polidori et al., 2000). However, practice time could induce anti-oxidant activity and improve inflammatory balance (Simioni et al., 2018). Moreover, resistance and aerobic training have been shown to increase the number of satellite cells, which may be necessary for regulating skeletal muscle TL (Kadi and Ponsot, 2010).

Studies systematically reviewing the effect of physical activity on TL concluded that physical activity seems to positively affect TL (Mundstock et al., 2015b; Arsenis et al., 2017; Lin et al., 2019). However, there is not enough evidence to fully elucidate the underlying mechanisms in detail from these studies. Another systematic review focused on the association between cardiorespiratory fitness and TL, and the evidence was also not sufficient to entirely understand this complex issue (Marques et al., 2020). Thus, the role of physical activity in combating the typical age-induced decrements in TL remains without a clear definition. Given the importance of TL, there is a need to understand better the potential association with another important component of health-related physical fitness, such as muscular fitness. Thus, this study systematically reviewed the evidence regarding the association between TL and muscular fitness in adults. Given the exposed mechanisms of TL, it is hypothesized that greater TL is associated with better muscular fitness.

## Methods

### Inclusion Criteria and Search Strategy

The review was performed following the Preferred Reporting Items for Systematic Reviews and Meta-Analyses (PRISMA) guidelines (Moher et al., 2009). Articles on the relationship between muscular fitness and TL published in peer-reviewed journals until the 31st of October 2020 served as a basis for this review. Eligibility criteria included the following: (a) cross-sectional, prospective, and experimental study design (study design criterion); (b) outcomes included TL (outcome measure criterion); (c) muscular fitness and TL (relationship criterion); (d) young, adults, and older adults (participants criterion); (e) articles published in English, Portuguese, or Spanish (language criterion); (f) articles were left out if they did not meet inclusion criteria or did not have findings associated to the inclusion criteria (exclusion criteria).

In October 2020, the article search was conducted in PubMed, Scopus, and Web of Science. Articles that assessed the relationship between muscular fitness and TL were included. The search was performed using the following terms: telomer^*^ AND fitness OR muscle^*^ OR muscular. The research team defined search terms, and the same terms were used in each database to identify articles for review. Two reviewers worked individually and screened titles and abstracts to identify articles that met the inclusion criteria. Duplicate entries were removed. Articles meeting the inclusion criteria were retrieved for a full read. Two authors reviewed the full text of potential studies, and decisions to include or exclude articles in the review were made by consensus. However, a third reviewer served as a judge to solve eventual disagreements.

### Data Extraction

The PRISMA statement (Moher et al., 2009) was used for data extraction. From each article, it was extracted the authors' name, year of publication, study design, sample characteristics, country, method of TL evaluation, methods of muscular fitness evaluation, study quality, and main results. The extraction was carried out by one author, and coding was verified by two other authors.

### Study Quality and Risk of Bias

The methodological quality of the articles was assessed by two researchers independently using the Physiotherapy Evidence Database (PEDro) scale. Agreement between reviewers was assessed using k statistics (*k* = 0.96) for full-text screening and rating of relevance and risk of bias. A third reviewer solved eventual disagreements about the risk of bias and made the final decision. The methodological quality of the included articles was assessed with a total score ranging from zero (lowest) to 11 (highest quality).

### Synthesis of Results

The review analyzed the relationship between muscular fitness and TL. Substantial heterogeneity existed across the reviewed studies for several study parameters. These parameters included: participant characteristics, tissue or fluid used to analyse telomeres, method of TL evaluation, and methods of muscular fitness evaluation. The details for each study, including design, measures, sample size and participant characteristics, and study quality and results, are presented consistently.

## Results

### Literature Search

**Figure 1:**
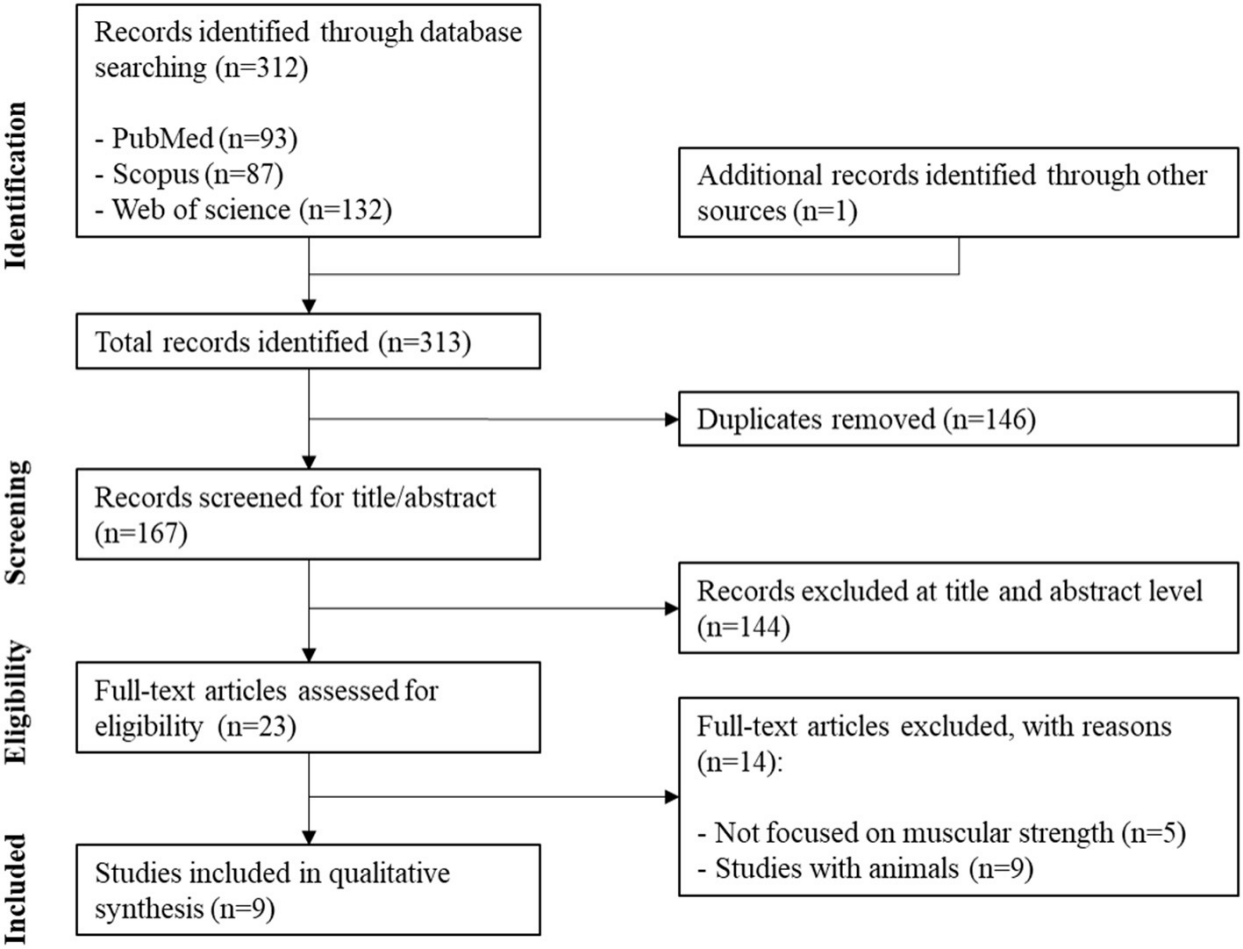
Flow diagram of study selection.

The search in the databases brought forth 312 records, and one record was identified through other sources. After excluding the 146 duplicates, 167 were selected for the title and abstract reading. Of these 167 articles, 24 records were discarded because not being research studies, seven were abstracts or letters to the editors, and 113 did not contain an assessment of muscular fitness and TL. From the 23 remained records, after reading the full text, 14 were discarded because five did not have a focus on muscular fitness and nine were studies with animals. Therefore, nine studies were finally included in the systematic review. The flow diagram is presented in Figure 1.

Table 1 presents the characteristics of the final set of studies. Nine studies were included in the review, totalling 20,269 adults, from six countries (China, Finland, South Korea, Sweden, United Kingdom, and the United States of America). Among the studies, four were cross-sectional, observational comparative studies, four were prospective studies, and one was a cross-sectional and a prospective observational study. The methods to assess TL were polymerase chain reaction (PCR) (6/9), Southern blot (2/9), and G-spin TM Genomic (1/9). The most frequent method used to evaluate muscular fitness was through maximal isometric grip strength (kg) measured by a hand dynamometer (7/9). Other measures of muscular fitness were the gait speed test (1/9) and powerlifting national completion performance (1/9).

**Table 1:** Characteristics and main results of studies included.

**References**	**Study design, sample characteristics (n, sex, age), country**	**Tissue or fluid; method of evaluation of telomeres**	**Evaluation of muscular fitness**	**Confounders adjusted for**	Study quality^*^	**Main results**
Kadi et al. (2008)	Cross-sectional observational comparative study. 14 men aged 28.56 ± 6.6 years (7 powerlifters who trained for 8 ± 3 years, seven healthy active subjects with no history of strength training). Sweden.	Peripheral leukocytes; Southern blot.	Squat and deadlift. All powerlifters participated in Swedish national competitions (7 ± 3 years). They had a training time of three to four sessions per week, corresponding to a mean of 7 h of training per week.	No information.	6	(±) Skeletal muscle DNA TL in powerlifters remained within the range of values found in subjects with no history of regular strength training.
Lee et al. (2013)	Cross-sectional observational study. 117 Korean elderly women, aged 74.2 ± 0.7 years. South Korea.	Blood; genomic DNA was extracted from whole blood using the G-spin TM Genomic DNA Extraction Kit.	Gait speed test. Participants were instructed to walk at a normal pace wearing comfortable shoes. The time was measured and gait speed was calculated as walking distance (6 m) divided by time.	Age, insulin resistance, and Mini-Mental State Examination.	5	(+) Leukocyte TL was independently associated with faster gait speed.
Baylis et al. (2014)	Prospective observational study, 10 years of follow up. 253 adults aged 67.1 ± 2.2 (158 men, 95 women). United Kingdom.	Blood; TL was measured as the ratio of the starting for telomeres vs. the starting for the single-copy gene of glyceraldehyde 3-phosphate dehydrogenase by real-time PCR.	Grip strength in the dominant hand was measured using a hand-held JAMAR dynamometer, recording the force (in kilograms).	Sex, age, height, weight for height, smoking, alcohol, social class, and cytomegalovirus seropositivity.	7	(+) Percentage change in TL over the follow-up period was significantly associated with greater grip strength at both unadjusted and adjusted analyses. Faster TL attrition was associated with lower grip strength at follow-up. However, this association was attenuated when adjusted for inflammation burden.
Woo et al. (2014)	Prospective, observational study, five years of follow up. 2006 participants (976 men, 1030 women) aged 72.4 ± 5.1 at the baseline. China.	Blood; quantitative PCR method was used to determine TL.	Grip strength in the dominant hand was measured using a hand-held JAMAR dynamometer, recording the force (in kilograms).	Age, education, body mass index, smoking, physical activity, and probable dementia.	7	(±) The association between TL and sarcopenia was not strong. However, longer TL was associated with a slower decline in grip strength in older persons.
Soares-Miranda et al. (2015)	Cross-sectional and prospective observational study. 582 older adults (221 men, 361 women), aged 73 ± five years at baseline in the Cardiovascular Health Study. United States of America.	Blood; TL was measured using Southern blot analysis.	Grip strength in the dominant hand was measured using a hand-held JAMAR dynamometer, recording the force (in kilograms).	Age, sex, race, education, income, smoking status, dietary habits, body mass index, fasting glucose, insulin, inflammatory markers and prevalent diseases.	6	(±) Grip strength was not significantly associated with TL in the prospective analysis.
Loprinzi and Loenneke (2016)	Cross-sectional observational study. 2410 adults aged 50–85 years participated in the 1999–2002 National Health and Nutrition Examination Survey (NHANES). United States of America.	Peripheral whole-blood; PCR/TS ratio.	A Kin Com MP dynamometer (Chattanooga Group, Inc.) was used to assess isokinetic knee extensor strength at peak force in newtons (at a speed of 60 degrees/second).	Age, sex, body mass index, diabetes, coronary artery disease, arterial pressure, smoking, and physical.	4	(+) Lower extremity muscular strength was associated with TL. The possible mechanism through which lower extremity muscular strength may be associated with morbidity and mortality.
Sillanpaä et al. (2017)	Cross-sectional observational comparative study. 11 women monozygotic twin pairs (age 57.6 ± 1.8 years) discordant for hormone replacement therapy. Mean duration of hormone replacement therapy among the users was 7.3 ± 3.7 years. Finland	Peripheral blood DNA by a quantitative real-time qPCR.	Vertical jumping height (cm) was calculated from flight time during a countermovement jump and measured on a contact mat. Grip strength was measured using an isometric dynamometer.	Age, physical activity, and hormone replacement therapy.	5	(+) There was no association between hormone replacement therapy use and TL. Skeletal muscle TL was associated with higher fat-free mass and greater thigh muscle area.
Williams et al. (2017)	Prospective birth cohort study (Northern Finland Birth Cohort 1966). The analyses were cross-sectional at the 31-year follow-up assessment. 5284 participants (2552 men, 2732 women), aged 31.2 ± 0.3 years. Finland.	Peripheral blood DNA by a quantitative real-time qPCR.	Maximal isometric grip strength (kg) of the dominant hand was measured by a hand dynamometer. Muscle endurance was assessed using a lower-back trunk muscle extension test. To measure aerobic fitness it was used the 4-min step test.	Age, sex, body mass index, socioeconomic position, diet quality, smoking, alcohol consumption, C-reactive protein, and physical activity.	7	(±) Longer TL was associated with higher aerobic fitness and trunk muscle endurance (but not with grip strength) in young adulthood.
Chang et al. (2020)	Prospective cohort study (Singapore Chines Health Study). Analysis were cross-sectional at a median 20-year follow-up assessment. 9581 participants (4061 men, 5520 women), aged 52.3 years. China.	Blood leukocyte TL by a quantitative real-time qPCR.	Maximal grip strength was calculated as the mean of the measurements from the right and left hands using a dynamometer.	No information.	7	(+) Longer mid-life blood leukocyte TL was associated with increased grip strength later in life.

*PCR, polymerase chain reaction; qPCR, quantitative-polymerase chain reaction; TL, telomere length*.

* *According to Physiotherapy Evidence Database (PEDro) scale*.

### Principal Findings

The main results from each study on the relationship between muscular fitness and TL are also presented in Table 1. The four prospective studies are mixed (Baylis et al., 2014; Woo et al., 2014; Williams et al., 2017). One observed that changes in TL over 10-years of follow-up were significantly associated with grip strength, and faster TL attrition was associated with lower grip strength (Baylis et al., 2014). Another study concluded that longer mid-life blood leukocyte TL was associated with increased grip strength later in life (Chang et al., 2020). However, in the other two prospective studies, the observed inverse association between TL and sarcopenia was not strong (Woo et al., 2014). Although longer LT was associated with higher aerobic fitness and trunk muscle endurance, it was not associated with grip strength in young adulthood (Williams et al., 2017). Nevertheless, longer TL was associated with a slower decline in grip strength in older persons (Woo et al., 2014). From the four cross-sectional studies, three indicated that leukocyte TL was associated with muscular fitness, and skeletal muscle TL was associated with higher fat-free mass and greater thigh muscle area (Lee et al., 2013; Loprinzi and Loenneke, 2016; Sillanpaä et al., 2017). On the other hand, in a cross-sectional study with powerlifters, TL remained within the range of values found in subjects with no history of regular strength training (Kadi et al., 2008), suggesting that muscular fitness was not associated with TL. The cross-sectional and prospective observational study showed that grip strength was not significantly associated with TL in the prospective analysis (Soares-Miranda et al., 2015).

## Discussion

Nine studies examining the association between muscular fitness and TL were identified. Overall, five studies reported that TL was positively associated with muscular fitness, including gait speed, grip strength, lower extremity muscular strength, fat-free mass, and thigh muscle area. On the other hand, the other four studies described inconclusive results.

TL shortening is associated with increased all-cause mortality risk (Wang et al., 2018) and limitations in physical function (Montiel Rojas et al., 2018). In turn, muscular fitness is positively associated with physical function and is negatively associated with all-cause mortality (Liu et al., 2014; Garcia-Hermoso et al., 2018). Accordingly, slightly more than half of the studies included in this review presented a positive association between TL and muscular fitness measures (Lee et al., 2013; Baylis et al., 2014; Loprinzi and Loenneke, 2016; Sillanpaä et al., 2017; Chang et al., 2020). These studies were mainly focused on middle-aged and older adults, strengthening evidence that points to TL being a biological marker of cellular senescence and physical aging (von Zglinicki and Martin-Ruiz, 2005). Especially among middle-aged and older adults, physical function is an important determinant of disease (O'Neill and Forman, 2020). Reinforcing this idea, in three of those studies, grip strength or gait speed were shown to be associated with TL (Lee et al., 2013; Baylis et al., 2014; Chang et al., 2020). On the other hand, two of the studies included in the review also focused on older adults, presented dissimilar findings, not sustaining the hypothesis of a direct association between TL and muscular fitness (Woo et al., 2014; Soares-Miranda et al., 2015).

It was observed that the association between TL and sarcopenia among Chinese older adults was not strong (Woo et al., 2014). Nevertheless, longer TL was still associated with a slower decline in grip strength. Also, among older adults from the USA, it was identified that grip strength was not directly associated with TL (Soares-Miranda et al., 2015). In summary, results from most studies performed in middle-aged and older adults seem to indicate a positive association between TL and muscular fitness, suggesting a possible connection between TL and healthy aging. Notwithstanding, more studies are warranted to better understand this association and the role of muscular fitness in the interaction with TL.

The shortening of TL is associated with cellular aging and can represent biological age (Arsenis et al., 2017). Thus, as people age, telomeres naturally shorten. However, their attrition is relatively stable from childhood to adulthood (Oeseburg et al., 2010). Two of the studies included in this review were conducted among younger adults (~30 years old), having presented non-significant or inconsistent findings (Kadi et al., 2008; Williams et al., 2017). In one study conducted in Sweden, it was found that the TL values of powerlifters (individuals regularly performing strength training) were similar to those with no history of regular strength training (Kadi et al., 2008). The second study, conducted in Finland, showed that longer TL was associated with higher aerobic fitness and trunk muscle endurance, but not grip strength (Williams et al., 2017). The interaction between TL and aging may explain these results among younger adults, as telomeres in younger ages are less likely to have experienced attrition. Therefore, behavioral factors such as physical inactivity and sedentariness (Arsenis et al., 2017).

Changing TL has been proposed to be one of the mechanisms through which muscular fitness is related to all-cause mortality and disease (Loprinzi and Loenneke, 2016). Mitochondrial dysfunction with age can in part explain the association between muscular fitness and TL, which is further supported by the relationship between sarcopenia, oxidative stress, and chronic inflammation (Marzetti et al., 2013). Notwithstanding, even when adjusting for inflammation (C-reactive protein) and antioxidant status (uric acid), the relationship between strength and TL remained significant (Loprinzi and Loenneke, 2016), suggesting that strength may influence TL through other mechanisms, which are still open to interpretation.

This review has some limitations that must be acknowledged and taken into account when interpreting the findings. Differences across the included studies (sample size, tissue sources, methods of evaluation of telomeres, and methods of muscular fitness evaluation) can influence the association between TL and muscular fitness and, therefore, may contribute to the variety of observed results. Also, some of the studies included in the review had small sample sizes. Nonetheless, the small number of empirical studies on the topic existing so far does not allow establishing a stratification of evidence by those additional factors. Additionally, the variety of study methodologies and outcome measures makes it impossible to adequately perform a meta-analysis.

The existing empirical evidence on the relationship between TL and muscular fitness is mixed and may be influenced by additional factors, such as individuals' age. In this regard, it seems that there is a positive association between TL and muscular fitness in middle-aged and older adults. However, among younger adults, such a relationship may not be evident.

## Data Availability Statement

The original contributions presented in the study are included in the article/supplementary material, further inquiries can be directed to the corresponding author.

## Author Contributions

AM, MP, DH-N, and AI: conceptualization. AM, MP, and DH-N: methodology development. MP and PM: formal analysis. MP and DH-N: investigation. AM, MP, and PM: writing original draft. EG, GF, JM, HS, and AI: writing review and editing. EG, GF, HS, and AI: visualization. HS and AI: supervision. All authors contributed to the article and approved the submitted version.

## Conflict of Interest

The authors declare that the research was conducted in the absence of any commercial or financial relationships that could be construed as a potential conflict of interest.
